# Sustainable Approaches for Carbon Powder-Filled ABS: A Comparative Study of Injection Moulding and Fused Filament Fabrication Technologies

**DOI:** 10.3390/polym17192593

**Published:** 2025-09-25

**Authors:** Vojtech Senkerik, Ales Mizera, Pavel Stoklasek, Lucie Svacinova, Lovre Krstulovic-Opara, Michaela Karhankova, Lukas Miskarik, Petra Bagavac, Miroslav Manas

**Affiliations:** 1 Faculty of Technology, Tomas Bata University in Zlin, 760 01 Zlin, Czech Republic; vsenkerik@utb.cz; 2 Faculty of Applied Informatics, Tomas Bata University in Zlin, 760 05 Zlin, Czech Republic; pstoklasek@utb.cz (P.S.); svacinova@utb.cz (L.S.); m_karhankova@utb.cz (M.K.); l_miskarik@utb.cz (L.M.); manas@utb.cz (M.M.); 3 Faculty of Electrical Engineering, Mechanical Engineering and Naval Architecture, University of Split, 21000 Split, Croatia; lovre.krstulovic-opara@fesb.hr (L.K.-O.); petra.bagavac@fesb.hr (P.B.)

**Keywords:** polymer recycling, carbon powder, acrylonitrile butadiene styrene (ABS), injection moulding, 3D printing, fused filament fabrication (FFF), composite materials, mechanical properties, sustainable manufacturing, tensile testing

## Abstract

The recycling of polymer composites remains a significant challenge due to both technical and economic obstacles. This study investigates the recycling potential of acrylonitrile butadiene styrene (ABS) composites filled with carbon powder (CP), employing injection moulding and fused filament fabrication (FFF) technologies. Laboratory-based experiments were conducted using ABS reinforced with 0.5, 1.0, and 1.5 wt.% CP to explore the tensile properties of mechanically recycled ABS+CP composites. The results indicate that CP addition positively influences tensile behaviour and that the ABS+CP composite maintains both tensile strength and stiffness after repeated processing. A concentration of 1.5 wt.% CP proved to be the optimal filler amount. The results for re-injection-moulded ABS + 1.5 wt.% CP demonstrate enhancements in tensile strength of approximately 3% and elastic modulus of approximately 15%, relative to virgin ABS. Similarly, such specimens reprocessed via FFF showed an average increase of 12% in tensile strength and of 27% in elastic modulus relative to virgin ABS across all three printing orientations (X, Y, and Z). These findings suggest improved interfacial adhesion and filler dispersion upon recycling. The study confirms the practical feasibility of ABS composite recycling and highlights their potential for structural and decorative use due to their appealing granite-like appearance.

## 1. Introduction

Additive manufacturing (AM), also commonly known as 3D printing, is a modern manufacturing technology that enables the creation of complex shaped parts directly from digital models without the need to modify production equipment. Based on the gradual addition of material layer by layer, this process offers significant time and material savings over traditional techniques such as milling or injection moulding. Thanks to its design flexibility, the ability to customize product properties, and the minimization of waste, 3D printing is finding widespread application in a variety of industries. Frequently used materials in this process include polymers, which can be modified and combined with different fillers to achieve specific properties depending on the application and printing method [1,2].

One of the most widely used 3D printing technologies is Fused Deposition Modelling (FDM), also known as Fused Filament Fabrication (FFF). This technology has gained popularity due to its low cost, ease of operation, and ability to process a wide range of materials. In the FFF process, polymers such as Acrylonitrile Butadiene Styrene (ABS), Polylactic Acid (PLA), or Polypropylene (PP) are melted and then deposited layer by layer using a nozzle to create the final object. In addition, this technology allows multiple materials to be printed simultaneously, working in multiple directions and creating large-scale parts. Although FFF is versatile and can process advanced materials such as biopolymers, hydrogel blends, or high-performance polymers such as Polyetheretherketone (PEEK), the resulting products often suffer from limited mechanical properties. Poor bonding between layers during printing leads to lower stiffness, toughness and thus limited use for applications requiring reliability and durability. This often restricts the use of 3D-printed polymer parts for prototyping and research. However, despite the mentioned disadvantages, the industry is showing growing interest in the use of 3D printing in the production of primary structural components [3,4,5,6].

To address the above challenges in 3D printing of polymer composites, recent studies have focused on both material innovation and process optimization to improve mechanical performance. Research has shown that modifying filament deposition strategies, such as aligning the grid orientation with the principal stress directions or using certain infill patterns, can significantly improve the fracture toughness and tensile properties of FFF-printed ABS components. Another material that can be 3D printed is High Density Polyethylene (HDPE), which excels especially in its chemical resistance and ease of processing. An additional advantage of this material is that the final products can be easily modified by radiation cross-linking, which significantly improves their temperature stability. In some cases, their mechanical properties are also improved [7,8,9,10,11,12,13,14].

Recent research has furthermore increasingly focused on polymer composites to overcome the inherent mechanical limitations of thermoplastics used in FFF, because polymer composites offer improved mechanical properties compared to conventional polymers. These composites are created by adding reinforcements to a thermoplastic polymer matrix, allowing their properties to be tailored to specific requirements. The reinforcements can take the form of particles (such as ceramic or metal), short fibres (such as glass or carbon fibres), continuous fibres, or nanomaterials (such as graphene or carbon nanotubes). Each type of reinforcement provides specific benefits such as higher strength, improved wear resistance, better thermal stability or electrical conductivity, making these materials suitable for advanced applications. Unfortunately, the processability of polymer composites in 3D printing is more difficult than that of pure polymers, mainly due to anisotropy of properties and fluctuation of materials during extrusion through the extruder nozzle [15,16,17,18,19,20,21].

Some properties of different types of composites have already been explored in previous research. The incorporation of short glass fibres (SGFs) into ABS matrices has been shown to significantly enhance mechanical performance, with improvements of over 50% in both tensile and flexural strength, and better structural integrity or surface quality of printed parts. In parallel, emerging fillers such as thermal black (TB), a high-purity carbon black alternative, have demonstrated promising results as eco-friendly additives, improving the electrical conductivity, thermal stability, and mechanical strength of ABS and Polyamide composites, while also reducing material costs and environmental impact [22,23,24,25,26].

Carbon fibres have gained considerable attention in additive manufacturing due to their exceptional strength-to-weight ratio, high stiffness, thermal stability, and electrical conductivity. These properties make carbon fibres particularly suitable for enhancing the mechanical performance of 3D-printed polymer composites, especially those based on ABS. The incorporation of short carbon fibres (SCF) into ABS matrices has demonstrated significant improvements in tensile strength, fracture toughness, and modulus, especially when the infill pattern is aligned with the principal stress direction. Moreover, carbon fibre-reinforced ABS (CF-ABS) composites exhibit enhanced interfacial bonding and reduced crack propagation, although porosity and fibre-matrix debonding remain challenges at higher print speeds and layer thickness. Hybrid composites combining carbon and glass fibres further leverage the advantages of both materials, offering superior flexural performance suitable for structural components in aerospace and automotive applications. The use of chopped carbon fibres also significantly enhances fracture resistance in mixed-mode loading, depending on the internal architecture and filler content. Collectively, these studies highlight the pivotal role of carbon fibre reinforcements in enabling the production of high-performance, multifunctional 3D-printed composites [27,28,29,30,31,32,33,34,35,36]. The influence of short carbon fibre (SCF) reinforcement on the mechanical properties of 3D-printed ABS produced via FFF was thoroughly investigated also by Lobov et al. [37]. In their study, they compared pure ABS with SCF-reinforced ABS using tensile, flexural, and fracture toughness tests at various production parameters (nozzle diameter, fill angle). It was demonstrated that orienting the fill in the direction of loading significantly increases the modulus of elasticity (by more than 1.7×) and tensile strength (by more than 1.5×). A larger nozzle diameter led to an average increase in tensile strength of 12.5%. On the other hand, when loaded perpendicular to the layers, the fibre-reinforced samples did not show a significant improvement over pure ABS, which was also confirmed by fracture tests. This work demonstrates that the combination of SCF reinforcement and optimized printing parameters can significantly enhance the performance of polymer composites in additive manufacturing [37].

Overall, advanced reviews highlight significant progress in the additive manufacturing of thermoplastic fibre-reinforced composites, with innovations in fibre alignment, printhead design, and processing strategies enabling higher fibre volume fractions and multifunctional capabilities. Together, these advancements underscore the potential of combining material innovation with process refinement to produce high-performance, functionally optimized components through additive manufacturing [38,39,40].

However, the current industry must focus not only on producing the highest-quality products while considering their cost, but also on sustainability. The trend is to move towards the production of so-called sustainable materials that will not burden the environment as existing or raw materials do. Therefore, it is necessary to consider various methods of recycling, i.e., the reuse of already manufactured products into new full-value products. However, the recycling of 3D-printed polymer composites, particularly those produced via FFF, presents a complex challenge due to both material and structural factors. While thermoplastic polymers like ABS can theoretically be reprocessed, a higher number of repeated thermal cycles tend to increase porosity and reduce mechanical performance, including tensile strength, strain at break, and overall toughness. Moreover, the incorporation of reinforcing fibres such as glass or carbon further complicates recycling efforts, as these materials often lead to heterogeneous blends that are difficult to remelt and process consistently. Certain studies suggest that blending recycled ABS with virgin material can partially restore mechanical properties and maintain print quality. However, instability of process parameters remains a concern, especially with increased recycling iterations. Additionally, current industrial recycling practices often struggle with contamination, inconsistent feedstock composition, and degradation of polymer chains, which collectively hinder the scalability of sustainable additive manufacturing. These limitations highlight the urgent need for improved recycling protocols, standardized material sorting systems, and innovative design strategies that prioritize recyclability from the outset [41,42,43,44,45,46,47,48].

García et al. [49] investigated the recycling potential of ABS using an injection moulding process, focusing on the influence of repeated recycling on the material’s mechanical properties. In the study, ABS was subjected to multiple cycles of grinding and reprocessing through injection moulding, with evaluations conducted after each cycle to measure changes in tensile properties. The study found that reprocessing ABS on an injection moulding machine did not significantly affect the processing properties until the third processing cycle. After the first and second recycling of ABS, there was an improvement in tensile properties, but after the third recycling, there was a decrease in tensile properties to the value of virgin ABS. This demonstrates that ABS can be effectively recycled while maintaining acceptable performance, supporting its use in sustainable manufacturing and waste reduction strategies [49].

Cress et al. [50] investigated the influence of multiple recycling cycles on the mechanical behaviour and structure of ABS parts manufactured by fused deposition modelling, revealing a clear degradation trend in tensile strength and ductility associated with increased porosity. Their findings highlighted that, although the intrinsic properties of the recycled filament remained relatively stable, the printing process itself introduced structural defects that significantly impacted the final part performance. The study further demonstrated that variations in porosity were the primary factor behind the stochastic nature of mechanical failure, as confirmed by Weibull statistical analysis. These insights underscore the necessity of optimizing FDM parameters, such as air gap and extrusion control, to minimize variability and enhance the mechanical reliability of recycled polymer components in additive manufacturing applications [50].

Vidakis et al. [51] investigated the mechanical behaviour of ABS polymer subjected to multiple mechanical recycling cycles in the context of fused filament fabrication, revealing a significant improvement in mechanical performance over a limited number of recycling repetitions. Through systematic testing—including tensile, compression, flexural, impact, and micro-hardness analyses—they observed that the mechanical properties of ABS increased progressively up to the fifth recycling cycle, with an average enhancement of approximately 30% across all measured characteristics between the third and fifth cycles. However, a marked deterioration of properties was detected after the fifth cycle, indicating a limit to the recyclability of the material without compromising structural integrity [51].

Based on an extensive literature review, particularly sourced from high-impact scientific journals, it has been established that ABS can be effectively reprocessed by injection moulding without significant deterioration of its tensile performance. Additionally, existing studies have confirmed that mechanically recycled ABS can be successfully utilized for the production of filament suitable for additive manufacturing (3D printing), yielding mechanical properties comparable to those of virgin ABS. However, despite these encouraging results, a notable gap remains concerning the sustainable processing of carbon-filled thermoplastic composites. Specifically, very limited research exists on the recycling or reuse of ABS reinforced with carbon powder, and currently, no simple, cost-effective technique has been proposed for converting these composite materials into high-quality secondary products. For example, study by Chawla et al. [52] investigated the use of recycled ABS filled with wood dust, Fe powder, and Bakelite powder for tile production through FFF technique with focus on various raster angles, infill density and speed. But, it did not include a direct comparison with pure ABS or first-processed ABS reinforced with fillers [52].

This research addresses precisely this serious gap, investigating the recycling and reuse potential of ABS composites containing carbon powder through both injection moulding and 3D printing processes. Special attention is devoted to systematic evaluation and comparison of mechanical behaviour, particularly the tensile properties, of both primary and secondary manufactured components, thereby contributing valuable insights towards enhancing the sustainability and circularity of filled thermoplastic materials.

## 2. Materials and Methods

### 2.1. Used Materials

In this study, a commercially produced Acrylonitrile Butadiene Styrene (ABS) with the trade name Novodur HH-112 was used, which was supplied by INEOS (London, UK). Due to its melt volume rate of 5.5 cm^3^/10 min (220 °C/10 kg), this polymer is easily processed by injection moulding technology, and it also stands out for its high surface quality and good impact strength. The material supplier recommends using this material in applications such as housings for electrical and electronic devices.

**Figure 1:**
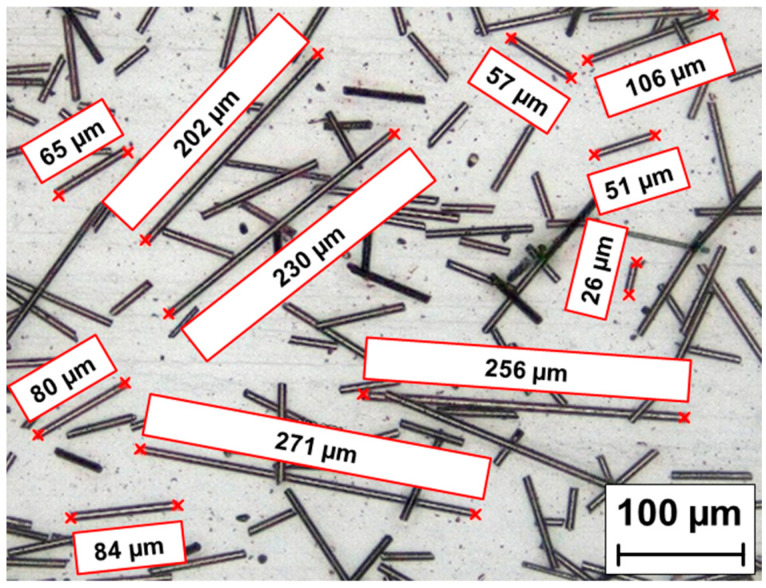
Particle length of the carbon powder (CP).

Commercially sourced carbon powder—CP (more specifically ground carbon fibres) supplied by Havel Composites CZ s.r.o. (Svesedlice, Czech Republic) were utilized as a filler material in the ABS matrix. The particle length of the CP ranged from approximately 25 µm to 275 µm, as illustrated in Figure 1. The incorporation of CP into polymer matrices is primarily aimed at enhancing mechanical properties, particularly tensile strength and stiffness. Due to its specific strength, carbon powder is especially advantageous for applications in automotive, aerospace, and high-performance sports equipment. Furthermore, the conductive nature of CP enables effective dissipation of electrostatic charges, a property that is important in environments where electrostatic discharge poses a risk to equipment or safety.

### 2.2. Specimen Preparation

**Figure 2:**
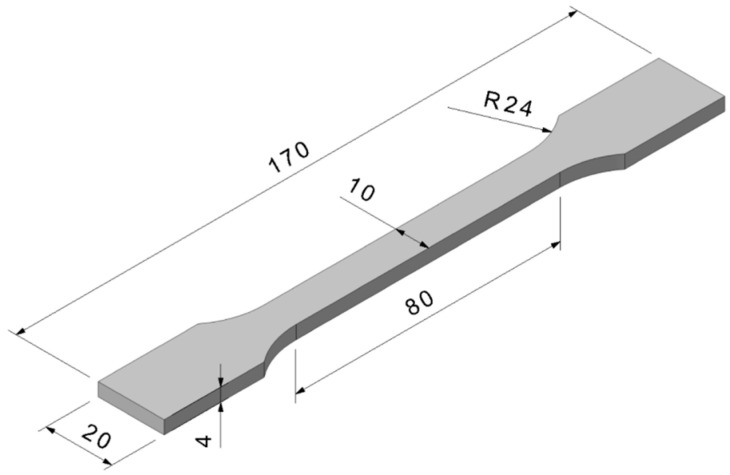
Shape and dimensions of tested specimens.

The preparation of test specimens (Figure 2) was conducted in accordance with the EN ISO 527-1 standard [53]. Initially, both unfilled (virgin) and carbon powder-filled ABS specimens were fabricated using an injection moulding process. The injection moulded specimens were subsequently crushed using a slow-speed knife mill with a sieve mesh size of 8 mm. The crushed carbon-filled material was then reprocessed using an injection moulding machine to produce re-injected specimens to simulate repeated heat treatment of the polymer composite. These re-injected specimens were again crushed to obtain ground material suitable for filament extrusion, which was subsequently used in the fused filament fabrication (FFF) process for 3D printing of the final test specimens.

#### 2.2.1. Injection Moulding

The first group of test specimens was composed of virgin ABS without any filler. The second through fourth groups comprised ABS compounded with 0.5 wt.%, 1.0 wt.%, and 1.5 wt.% of carbon powder (CP), respectively. The polymer-filler mixtures were homogenized using a slow-speed paddle stirrer for 60 s to ensure uniform dispersion of the carbon powder within the ABS matrix. The fifth through seventh groups were prepared from injection moulded ABS specimens identical in initial composition to the second through fourth groups (i.e., containing 0.5 wt.%, 1.0 wt.%, and 1.5 wt.% CP, respectively). These specimens were subsequently subjected to a grinding process using an SPGL 1830X knife mill (APplastic, s.r.o., Třinec, Czechia), and the resulting regrind material was reprocessed through injection moulding to produce re-injected specimen groups number five to seven, enabling the assessment of the effects of repeated processing on material properties. For clarity, Table 1 shows the identification of the specimen groups with a brief description that summarizes each specimen group.

Prior to each injection moulding process, both the virgin ABS granulate and the ABS with carbon powder (ABS+CP) were dried at 80 °C for 4 h using an Arburg Thermolift 100-3 granulate dryer (Loßburg, Germany) to minimize moisture-related defects during processing. All test specimens were fabricated via injection moulding on an Arburg Allrounder 370 S 700-100 machine, equipped with a 30 mm screw diameter (Loßburg, Germany). The injection moulding parameters were optimized in accordance with the manufacturer’s recommendations (INEOS Styrolution Group GmbH, London, UK), as detailed in Table 2.

#### 2.2.2. Filament Extrusion

The filament was produced using two types of materials: virgin ABS and ground material obtained from three groups of re-injected composites. Namely, the fifth group (re-injected ABS+0.5 wt.% CP), the sixth group (re-injected ABS+1.0 wt.% CP), and the seventh group (re-injected ABS+1.5 wt.% CP). In order to create a granulate suitable for extrusion, these injected specimens were subjected to mechanical size reduction, i.e., ground using an SPGL 1830X knife mill (APplastic, s.r.o., Třinec, Czechia). The ground materials were dried prior to extrusion at 80 °C for 4 h using an Arburg Thermolift 100-3 granulate dryer (Loßburg, Germany) to eliminate residual moisture.

For the fabrication of 3D printing filament, the Composer 450 filament extruder (3devo, Utrecht, Netherlands) was selected. This compact, all-in-one system is specifically designed for the laboratory-scale production of thermoplastic filament. The Composer 450 model is equipped with a mixing auger, which enhances the homogeneity of the polymer melt and ensures uniform dispersion of additives and fillers within the matrix [18].

The extrusion system features a hardened screw manufactured from a high chromium and molybdenum alloy steel, housed within a barrel divided into four independently controlled heating zones. The processing temperatures for each zone were optimized for the extrusion of both virgin ABS and ABS with carbon powder (ABS+CP), as specified in Table 3.

To ensure consistent cooling of the extruded filament; the system incorporates a dual-position fan array, allowing for even air distribution along the extrusion path. The fan speed was set to 55% of its maximum capacity. Filament diameter monitoring is performed by an optical diameter sensor, capable of detecting diameters in the range of 0.5 mm to 3.0 mm. Directly beneath the sensor, an adaptive puller system continuously adjusts the pulling rate to maintain the target filament diameter with high precision

**Figure 3:**
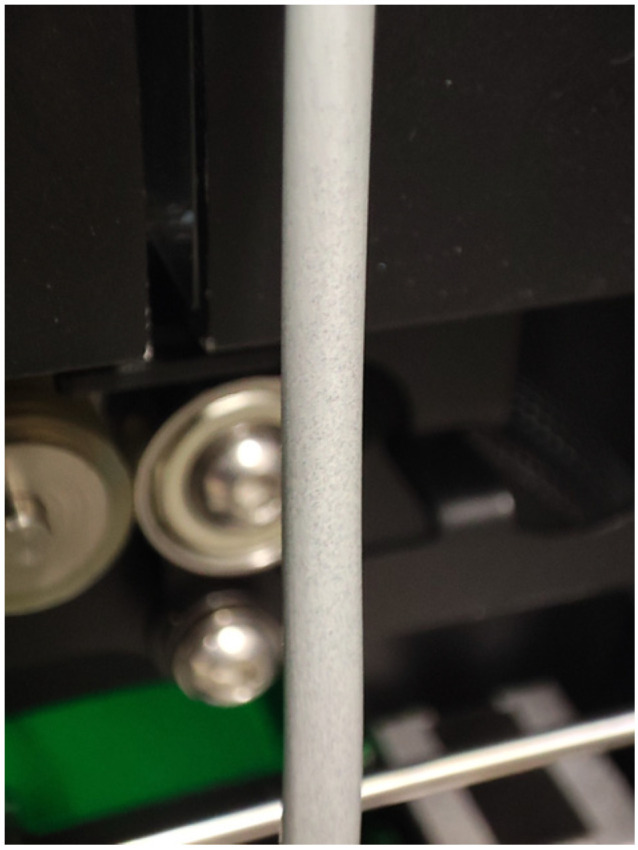
Shape of the extruded filament (before pulling).

Following the extrusion (Figure 3) and diameter stabilization phase, the filament passes through a filament positioner, which automatically guides it onto a pre-mounted spool. The spooling process is controlled by predefined parameters, ensuring uniform and organized winding of the filament.

All key extrusion parameters—including zone temperatures, screw rotation speed, fan cooling intensity, and filament diameter—were configured directly on the machine interface. A 4 mm extrusion nozzle was employed. Once the heating zones reached the target temperatures, the screw was activated at the set speed, initiating the extrusion process. The target filament diameter was set to 1.75 mm, and the screw rotation speed was maintained at 4 rpm throughout the extrusion process.

The extruded filament was directed through the optical diameter sensor and subsequently engaged by the adaptive puller system. A brief delay followed, during which the pulling speed was automatically adjusted to achieve the target filament diameter. Upon completion of the pulling calibration, the filament was guided through the positioner and affixed to the pre-mounted spool within the winding unit, ensuring controlled and uniform spooling.

#### 2.2.3. Three-Dimensional Printing

**Figure 4:**
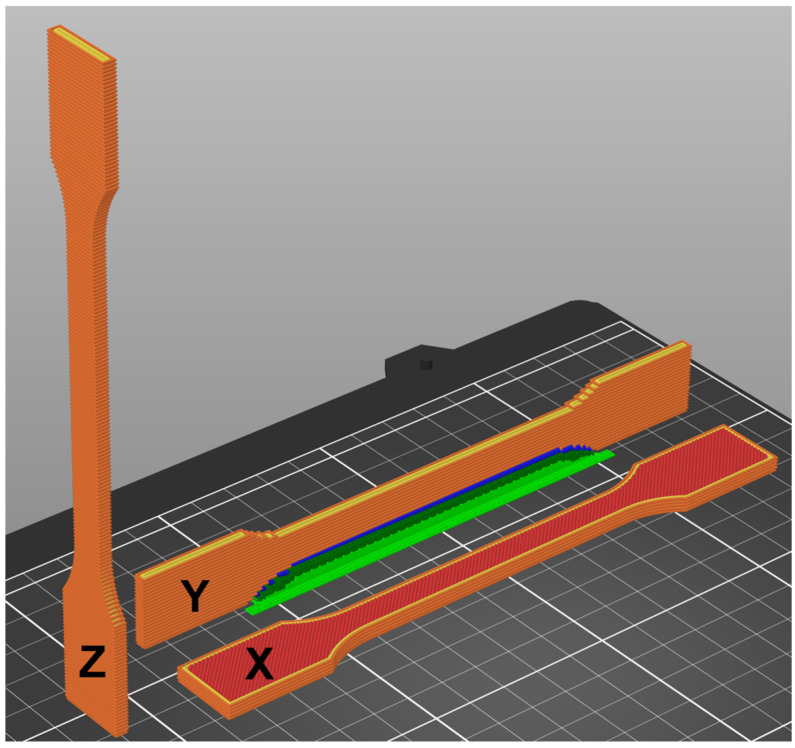
Specimens’ 3D-printed orientations.

Standardized tensile test specimens were fabricated from the prepared filaments using fused filament fabrication (FFF). A Prusa MK4 3D printer (Prusa Research, Prague, Czech Republic) equipped with a 0.4 mm brass nozzle was employed for this purpose. Specimens were printed in three orientations: X, Y, and Z as shown in Figure 4. The slicing and print preparation were conducted using PrusaSlicer (Prusa Research, Prague, Czech Republic), utilizing the default Generic ABS material profile. A predefined print mode labelled “0.2 mm Quality” was selected, which provides a balance between surface resolution and mechanical fidelity. To ensure maximal material density and minimize internal defects, the infill density was set to 100%, thereby producing fully solid parts suitable for mechanical testing.

The printing process adhered to the default temperature and speed parameters established within the slicing software for the selected ABS profile, ensuring consistency and reproducibility. The extrusion temperature was maintained at 260 °C, which lies within the optimal processing range for ABS to ensure proper interlayer bonding and filament flow. Simultaneously, the build plate temperature was set to 110 °C, facilitating adhesion during the initial layers and reducing the risk of warping or delamination during cooling.

These conditions reflect typical industrial standards for processing ABS via FFF, where thermomechanical stability and dimensional accuracy are essential. The use of manufacturer-recommended settings minimizes the influence of uncontrolled variables and enhances the reliability of intercomparisons across specimen groups. Moreover, the use of a high-quality printer such as the Prusa MK4, in conjunction with a carefully calibrated slicing profile, contributes to the reproducibility and mechanical integrity of the printed specimens used in subsequent material characterization.

### 2.3. Methods of Testing

The evaluation of tensile properties is a fundamental aspect of the mechanical characterization of polymer materials, especially in the context of additive manufacturing and materials processing. In this study, tensile tests were performed on test specimens in three phases of the tested material life cycle. First, injection moulded specimens were tested to establish a baseline reference value for the mechanical behaviour of the test specimens under standardized conditions. Second, the tensile properties of prepared filaments were measured to investigate the potential influence of filament extrusion on material integrity. Finally, specimens produced by fused filament fabrication (FFF) were tested in three main print orientations (X, Y, and Z) to investigate the anisotropic nature of printed parts. This multifaceted approach allows for a detailed comparison of tensile properties across different processing methods throughout the life cycle of ABS products reinforced with carbon powder.

#### 2.3.1. Tensile Properties

**Figure 5:**
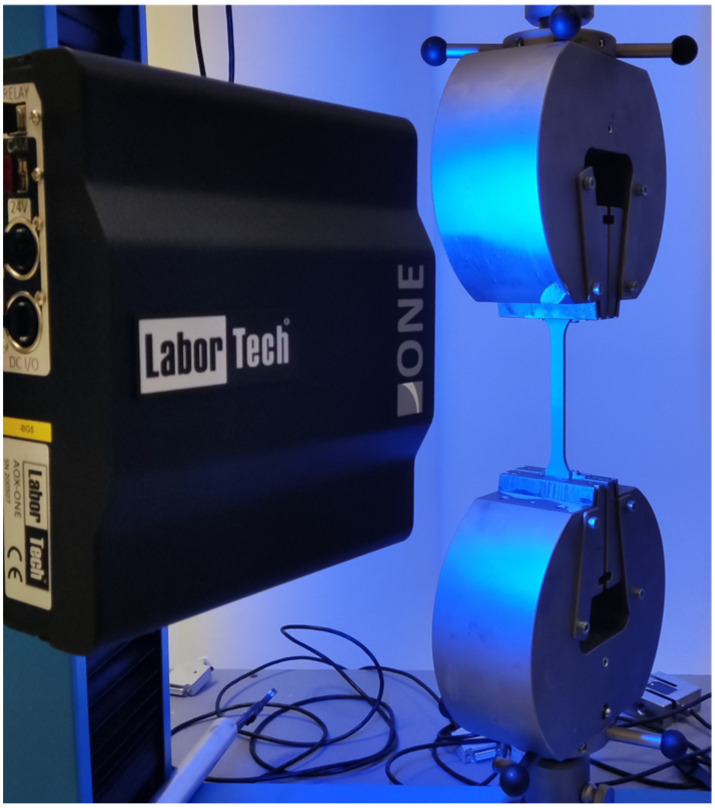
Hardware configuration according to ISO 527 standard.

Tensile properties were evaluated using a LabTest 6.50.1.10 uniaxial electromechanical testing machine (Labortech, Opava, Czech Republic), which was configured in two distinct hardware setups to accommodate the requirements of the study. The first configuration (Figure 5) adhered to the ISO 527 standard [53] for tensile testing of plastics and was performed under controlled ambient conditions (23 °C and 60% relative humidity). In this setup, standard dog-bone specimens (Figure 2) produced by both injection moulding and fused filament fabrication (FFF) were subjected to uniaxial tensile loading at a constant crosshead speed of 50 mm/min. A ONE optical extensometer was employed to capture precise strain measurements during testing. Five specimens were tested from the injection moulded group and three specimens from the 3D-printed group. The primary mechanical parameters assessed included the elastic modulus (Young’s modulus) and tensile strength, providing a quantitative basis for comparing the mechanical performance across manufacturing methods.

**Figure 6:**
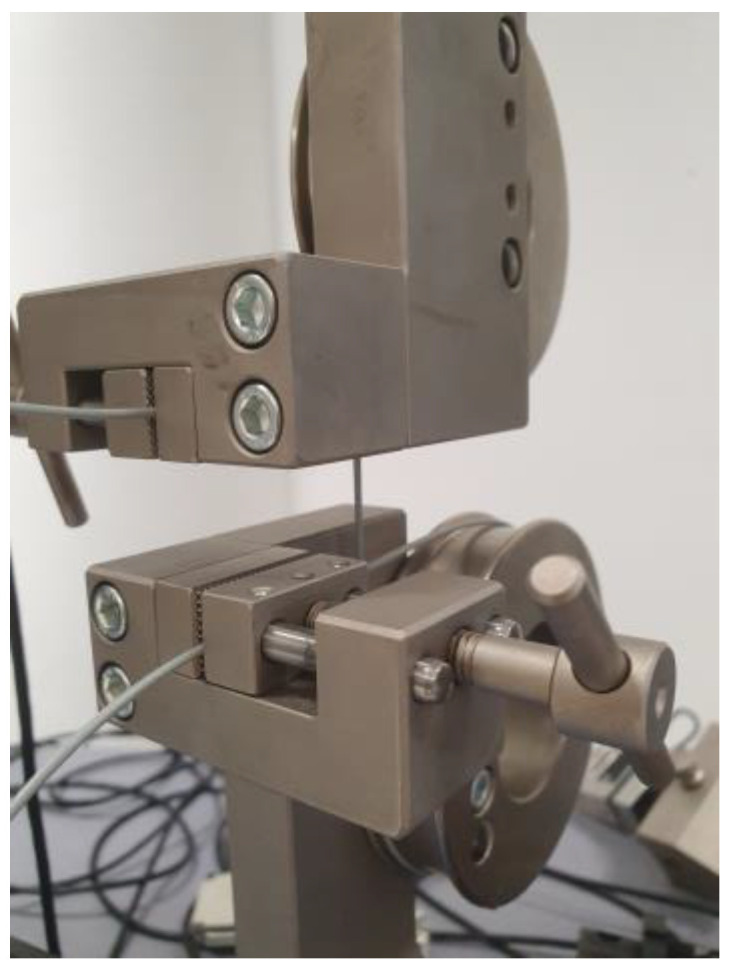
Hardware configuration for measuring filaments.

The second experimental configuration (Figure 6) was specifically designed to evaluate the tensile properties of the filaments. In this setup, the axes distance between the belt wheels was fixed at 130 mm to ensure consistent gauge length across all tested samples. The tensile tests were conducted at a constant crosshead speed of 50 mm/min, identical to that employed in the first configuration, thereby ensuring methodological consistency. Ten specimens were tested from each filament sample group.

Prior to testing, all specimens and filaments were conditioned for a period of five days under controlled environmental conditions of 23 °C and 60% relative humidity, in accordance with standard practice for polymer testing. To enhance the clarity and reproducibility of the reported data, all quantitative results are presented as arithmetic means accompanied by their respective standard deviations.

#### 2.3.2. Evaluation of Fracture Surfaces

Fractographic and microscopic analyses of the tested specimens were performed using a Keyence VHX-6000 digital microscope and a Keyence VK-X1000 confocal laser scanning microscope (Mechelen, Belgium). These advanced imaging systems enabled high-resolution visualization and detailed optical characterization of the fracture surfaces. The analyses were conducted after the tensile testing to ensure a direct correlation between mechanical failure and the underlying fracture mechanisms. Particular emphasis was placed on identifying surface topographies and morphological features indicative of the material’s failure behaviour. This comprehensive approach provided valuable insights into the microstructural responses associated with different processing conditions and loading orientations.

## 3. Results

The object of this research was to investigate the tensile properties of repeatedly processed (mechanically recycled) ABS+CP composites. The aim was to determine to what extent these properties differ from virgin mechanically recycled ABS, as well as the effect of different ratios of added CP and the effect of different processing methods. The values of tensile strength (σₘ) and elastic modulus (E), both expressed in MPa, were measured in this testing.

### 3.1. Tensile Properties of Injection Moulded Specimens

First, the values of the injection moulded specimens were measured, comparing first injection moulded virgin ABS without any filler (G1) with groups of composite specimens, as listed in Table 1. Groups G2 to G4 were composed of first-injected ABS reinforced with CP at concentrations of 0.5 wt.%, 1.0 wt.%, and 1.5 wt.%, respectively. Groups G5 to G7 were manufactured from re-injected compound of ABS reinforced with CP in concentrations of 0.5 wt.%, 1.0 wt.% and 1.5 wt.%, respectively.

**Figure 7:**
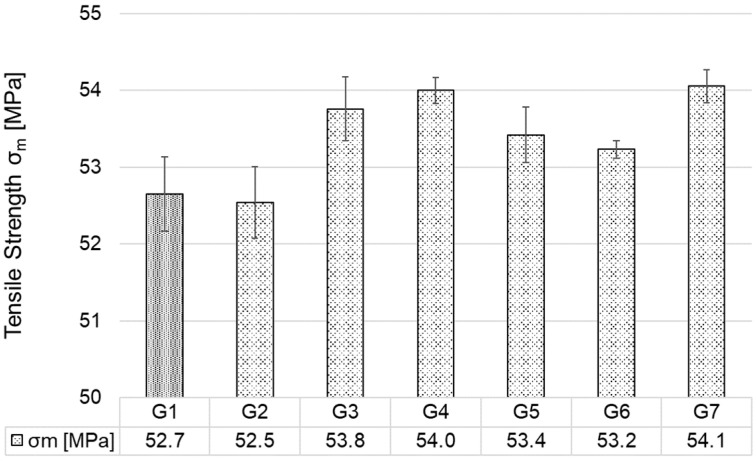
Tensile strength of injection moulded specimens.

Figure 7 graphically shows the results of the arithmetic mean of the tensile strength values of all injection moulded test specimens (G1–G7). The addition of 0.5 wt.% CP to ABS (G2) resulted in a negligible decrease in tensile strength after the first injection—the values lie within the standard deviation of values G1 and G2. However, an increase in tensile strength was observed in all other groups G3–G7. In group G5, re-injection moulding resulted in an increase in tensile strength of approximately 1.5% compared to group G2 and group G1. As confirmed by Cress et al. [50] and Vidakis et al. [51], mechanical recycling with a low number of reprocessing cycles can have a positive effect on the tensile properties of ABS. The best tensile strength values were achieved in group G7—approximately 3% higher than G1. From the measurement can be concluded that the addition of CP leads to a slight increase in tensile strength with increasing CP content up to a used value of 1.5 wt.%. As for reprocessing (specimen groups G5–G7), there was no significant decrease compared to the primarily processed specimens of groups G2–G4; in fact, two groups showed improved results.

**Figure 8:**
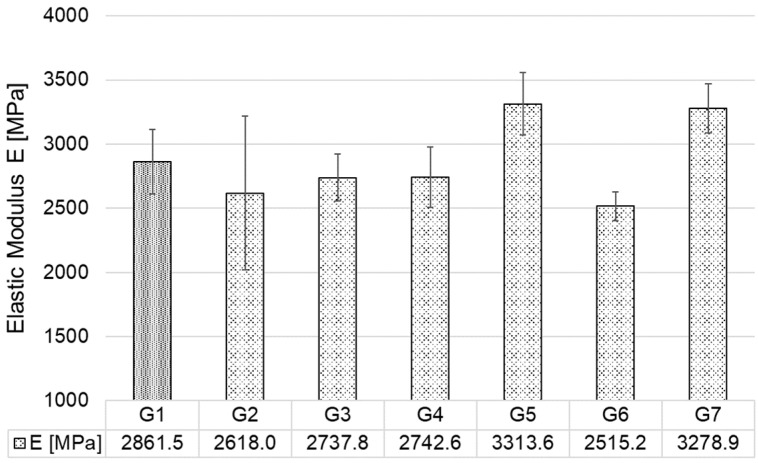
Elastic modulus of injection moulded specimens.

Figure 8 graphically displays the results of the arithmetic mean of the elastic modulus values of all injection-moulded test specimens (G1–G7). For the first injection moulded ABS test specimens with CP content of 0.5, 1.0, and 1.5 wt.% (G2–G4), there was a decrease in elastic modulus of 6% on average compared to G1. After re-injection, there was a further decrease in elastic modulus of nearly 9% in specimens G6 compared to G3. However, a significant increase in elastic modulus was achieved in groups G5 and G7, approximately 15% compared to G1.

Based on the tensile properties in terms of tensile strength and elastic modulus, it can be concluded that mechanical recycling of ABS reinforced with CP can also have a positive effect, especially when filling ABS with 1.5 wt.% CP.

### 3.2. Tensile Properties of Prepared Filaments

**Figure 9:**
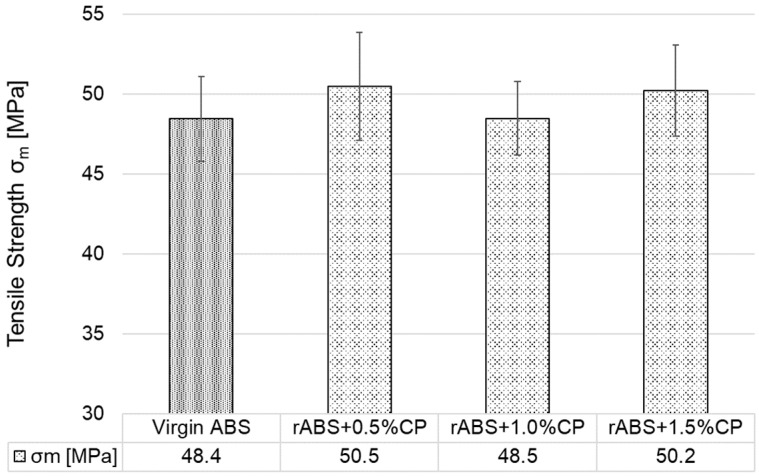
Tensile strength of prepared filaments.

Secondly, the mechanical properties of the extruded filaments were systematically evaluated, with the tensile strength results summarized in Figure 9. The filaments were fabricated using either virgin ABS granulate or reprocessed material from re-injected ABS composites containing CP. To improve clarity, horizontal axis labels in graphs were shortened and the weight percent indication was omitted.

The data indicate that the incorporation of CP, irrespective of concentration, resulted in a slight enhancement of tensile strength relative to the unmodified ABS baseline. Notably, the filament containing 1.0 wt.% CP demonstrated only a marginal improvement in tensile strength, amounting to merely a few tenths of a percent. In contrast, the specimens reinforced with 0.5 and 1.5 wt.% CP exhibited the most pronounced improvement, achieving a tensile strength approximately 4% higher than that of the virgin ABS; however, it can be observed that the values lie within the measurement error range. These findings underscore the potential of low-level CP reinforcement in optimizing the mechanical performance of recycled ABS filaments.

The results of the tensile testing conducted on the prepared filaments demonstrate that recycled ABS materials incorporating CP in concentrations up to 1.5 wt.% can be effectively utilized in additive manufacturing processes without any observable limitations. These findings confirm the suitability of such recycled composites for 3D printing applications, both in terms of mechanical integrity and processability.

### 3.3. Tensile Properties of 3D-Printed Specimens

Finally, to comprehensively evaluate the mechanical properties of the prepared filaments, the tensile properties of 3D-printed specimens were investigated. Specifically, the tensile strength and elastic modulus of specimens made from virgin ABS, rABS+0.5%CP, rABS+1.0%CP and rABS+1.5%CP were determined. The specimens were printed in three different print orientations to capture the anisotropic behaviour inherent in fused filament fabrication (FFF) processes, as shown in Figure 4. This analysis provides crucial insight into the structural integrity and stiffness of composite materials and elucidates the influence of CP reinforcement and print orientation on the tensile response.

#### 3.3.1. Orientation X

Fused Filament Fabrication (FFF) inherently exhibits pronounced anisotropy in mechanical properties, primarily due to the layer-by-layer deposition process and resultant orientation of the printed structures. Consequently, careful consideration must be given to the orientation of parts during the printing process, particularly with respect to the principal loading direction and the strategic placement of support structures. In practice, the most commonly employed orientation involves positioning the part on the largest possible surface area in contact with the build platform—an approach that, in this study, is designated as the X orientation.

**Figure 10:**
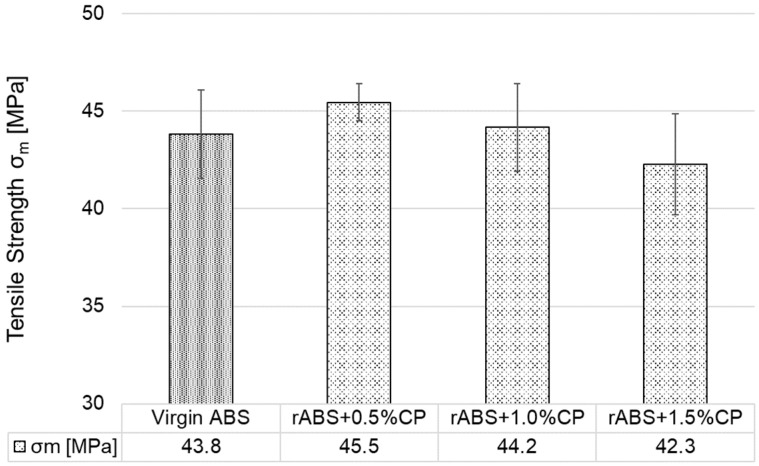
Tensile strength of 3D-printed specimens in orientation X.

Figure 10 presents the tensile strength (σₘ) of 3D-printed specimens fabricated from virgin ABS and re-processed ABS+CP composites reinforced with varying concentrations (0.5–1.5 wt.%) of carbon powder (CP), all printed in the X-orientation. The results demonstrate that the composite material, subjected to multiple processing cycles, retains its suitability for FFF 3D printing, comparable to that of virgin ABS. Notably, the rABS+0.5%CP formulation exhibited an improvement in tensile strength of nearly 4% relative to virgin ABS, indicating a beneficial reinforcing effect at lower CP concentrations. Then, a gradual increase in CP content led to a slight reduction in tensile performance, with the rABS+1.5%CP composite showing a modest decrease of approximately 3% in tensile strength compared to virgin ABS. These findings can suggest an optimal threshold for CP addition beyond which mechanical benefits begin to diminish. However, these measured differences in tensile strength are within the margin of error. This indicates that the addition of low-CP content does not cause any deterioration in tensile strength in the orientation X.

**Figure 11:**
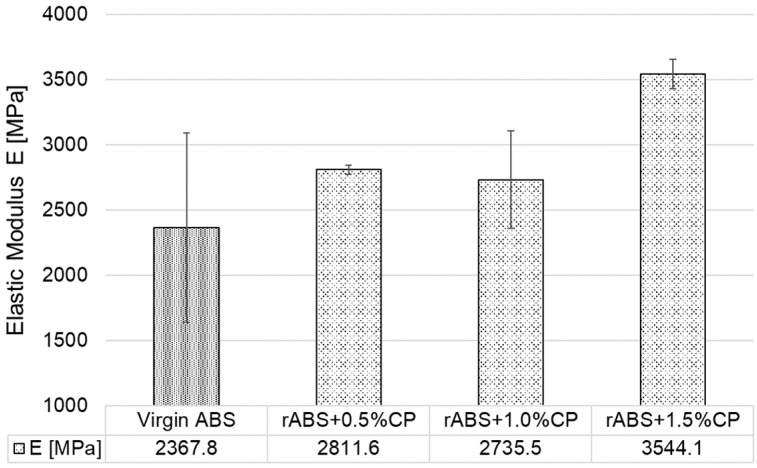
Elastic Modulus of 3D-printed specimens in orientation X.

Figure 11 illustrates the relationship between carbon powder (CP) concentration and the elastic modulus of 3D-printed specimens fabricated in the X orientation. The incorporation of a small amount of CP led to a notable enhancement in stiffness, with a significant increase in elastic modulus of nearly 20% observed at the lowest concentration. At the highest tested concentration of 1.5 wt.% CP, the elastic modulus exhibited an increase of approximately 50% relative to virgin ABS. This substantial improvement in stiffness underscores the pronounced influence of CP content on the mechanical behaviour of the printed components, with potential implications for their functional performance and load-bearing capacity in practical applications.

The incorporation of carbon powder into ABS matrices enables the reprocessing and production of 3D-printed components with mechanical properties comparable to, or even surpassing, those of virgin ABS, particularly at lower filler concentrations. While tensile strength reaches its peak at 0.5 wt.% CP, the elastic modulus continues to increase with higher CP content, indicating enhanced stiffness but also suggesting a trade-off between strength and rigidity at elevated filler levels.

#### 3.3.2. Orientation Y

In orientation Y, the printed layers are arranged parallel to the direction of tensile load, but perpendicular to the orientation of the main surface of the X orientation. The difference between the X and Y configurations is apparent in the shape of the base of the printed part. In this study, the test specimen has a functional part dimension of 10 mm in the orientation X, 4 mm in the orientation Y, and 170 mm in the orientation Z, which mainly results in differences in the application of the internal filling of the test specimen. This also affects the application of individual layers of material on top of each other. The result is anisotropic behaviour, which can significantly influence the mechanical performance of these printed parts.

**Figure 12:**
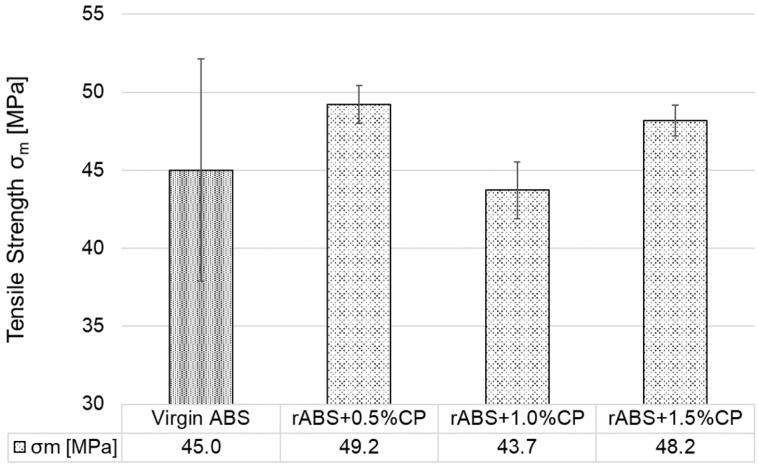
Tensile strength of 3D-printed specimens in orientation Y.

As shown in Figure 12, the tensile strength of specimens printed in the orientation Y predominantly exhibited a significant increase compared to the orientation X. The highest tensile strengths in the orientation Y were exhibited by the 3D-printed composites rABS+0.5%CP and rABS+1.5%CP, with an increase in tensile strength of 8% and 14%, respectively, compared to corresponding specimens in orientation X. The rABS+1.0%CP composite showed a slight decrease in tensile strength of approximately 3% compared to virgin ABS in the same orientation (Y). However, from the perspective of standard deviation, it can be observed that recycling ABS composite with carbon powder at a concentration of up to 1.5 wt. % does not cause a significant deterioration in tensile strength; on the contrary, in certain cases, tensile strength increases.

**Figure 13:**
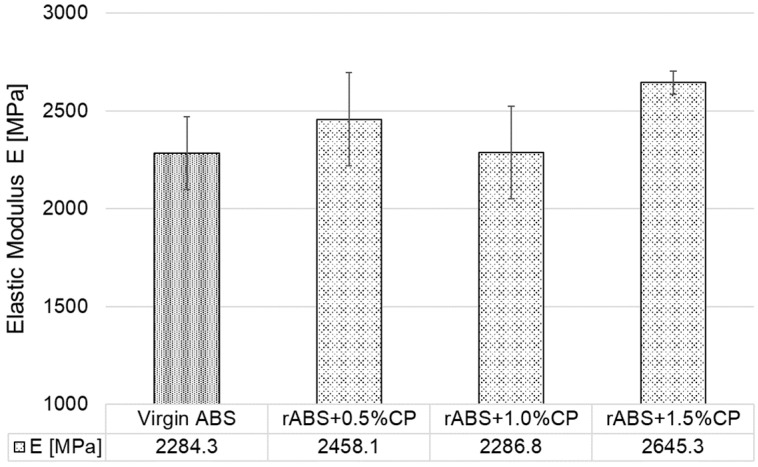
Elastic modulus of 3D-printed specimens in orientation Y.

The results of the elastic modulus shown in Figure 13 reflect a consistent trend with the results observed in the orientation X. With increasing CP content, a gradual increase in stiffness was observed, except for the composite labelled rABS+1.0%CP, which achieved approximately the same values as virgin ABS. The rABS+0.5%CP sample showed an 8% increase in elastic modulus compared to virgin ABS, while the sample with 1.5% wt. CP showed an almost 16% increase in modulus of elasticity. These results confirm that carbon powder can act as an effective reinforcing agent.

Overall, the mechanical response in the orientation Y shows a balance between maintaining tensile strength and improving stiffness. The data suggest that both tensile strength and elastic modulus do not deteriorate after the addition of CP; on the contrary, at CP concentrations of 0.5% and 1.5%, there was a significant improvement in both tensile strength and elastic modulus compared to virgin ABS.

#### 3.3.3. Orientation Z

The orientation Z corresponds to tensile loading applied perpendicular to the printed layers, thereby subjecting the interlayer bonds to maximum stress. This orientation is typically the most mechanically vulnerable due to the lower strength of layer adhesion compared to intralayer cohesion.

**Figure 14:**
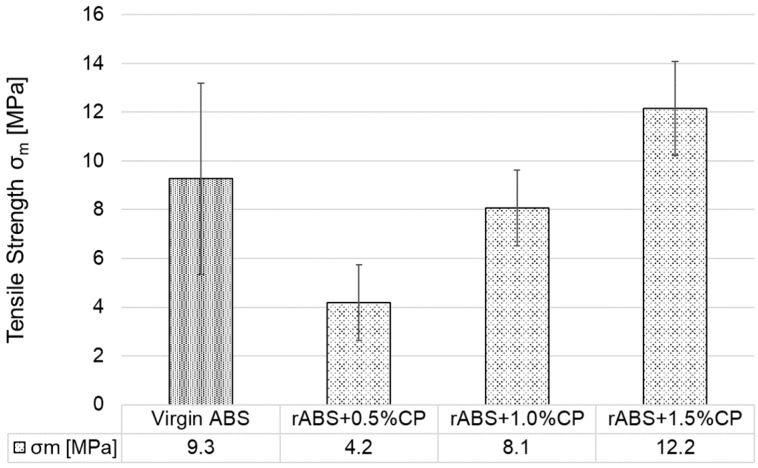
Tensile strength of 3D-printed specimens in orientation Z.

As illustrated in Figure 14, all tested specimens exhibited significantly reduced tensile strength in the orientation Z relative to the orientations X and Y. Although the composite rABS+1.5%CP exhibited the highest tensile strength within this group, it still showed a rounded reduction of 75% compared to its Y-oriented counterpart. The lowest tensile strength was achieved by the rABS+0.5%CP composite, which showed values reduced by half compared to virgin ABS in the orientation Z. When the concentration was increased to 1.0 wt. %, the tensile strength values were closer to the values of virgin ABS. Measurements have shown that if the composite is filled with CP, it is necessary to add at least 1 wt. % CP to ABS so that the strength in the least resistant Z orientation is not compromised.

**Figure 15:**
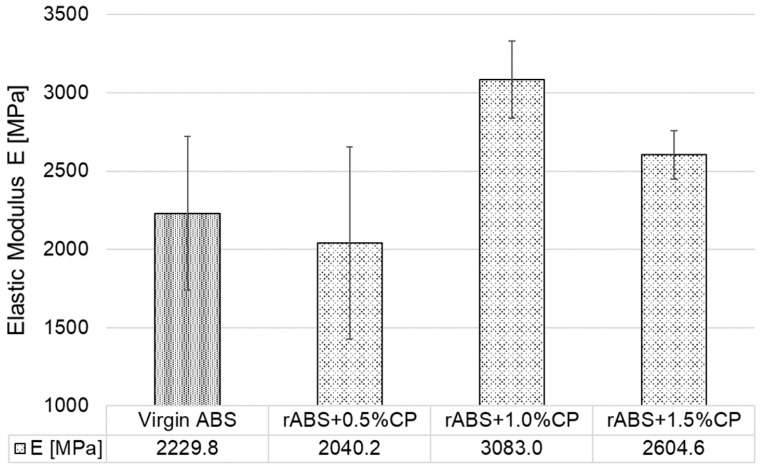
Elastic Modulus of 3D-printed specimens in orientation Z.

Figure 15 presents the elastic modulus (E) values of 3D-printed specimens in the Z orientation, fabricated from virgin ABS or reprocessed rABS+CP composites. The data reveal a nonlinear relationship between CP content and material stiffness. Virgin ABS exhibited an average elastic modulus of 2229.8 MPa. Interestingly, the addition of 0.5 wt.% CP resulted in a slight decrease in elastic modulus by almost 9% compared to virgin ABS, potentially due to weaker interfacial bonding. A substantial increase in stiffness was observed at 1.0 wt.% CP, where the elastic modulus reached a value approximately 40% higher than that of virgin ABS—indicating a reinforcing effect at this concentration. Further increasing the CP content to 1.5 wt.% led to a moderate decline in elastic modulus to 2604.6 MPa, though still representing an improvement of roughly 17% compared to virgin ABS. The relatively large error bars, particularly for virgin ABS and rABS+0.5%CP, suggest a higher variability in mechanical response, likely arising from inconsistencies in interlayer adhesion. Overall, the results for orientation Z indicate that a CP concentration of 1.0 wt.% provides an optimal balance for enhancing stiffness in rABS based composites.

In summary, the orientation Z underscores the anisotropic nature of FFF fabricated materials. The results emphasize the importance of optimizing both material composition and print orientation to achieve desirable mechanical properties in 3D-printed components. Low content of CP can improve stiffness and strength across all orientations. However, it is necessary to thoroughly test these new materials to prevent unexpected failure of 3D-printed parts due to poor design of both the material and the structure.

### 3.4. Evaluation of Fracture Surfaces

Fractographic and microscopic evaluations of the tested specimens were performed using a Keyence VK-X1000 confocal laser scanning microscope and a Keyence VHX-6000 digital microscope (Mechelen, Belgium). These advanced imaging systems enabled high-resolution observation and accurate optical characterization of fracture surfaces after tensile testing. The analyses focused on establishing a direct correlation between the observed fracture mechanisms and the mechanical properties of the materials. Special attention was paid to identifying surface topographies and morphological features that reflect the dominant fracture mechanisms.

#### 3.4.1. Injection Moulded Specimens Fracture Surface Evaluation

**Figure 16:**
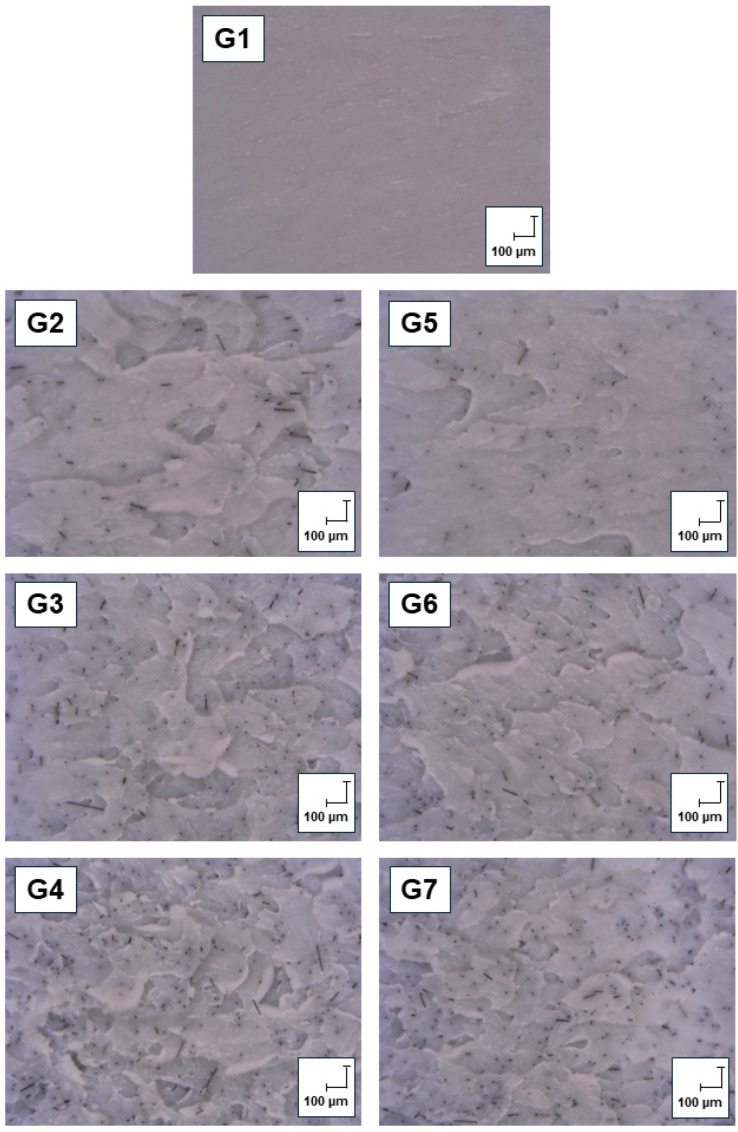
Fracture surface of a selected injection moulded specimens.

Figure 16 presents optical micrographs of the fracture surfaces of selected injection moulded specimens, corresponding to Groups G1 through G7, captured at identical 200× magnification with a scale bar of 100 µm for reference. Group G1, representing the virgin ABS, exhibits a relatively smooth and homogeneous fracture surface with minimal morphological features. Groups G2 to G4 show fracture surfaces of specimens with increasing concentrations (0.5, 1.0, and 1.5 wt.%, respectively) of carbon powder (CP). These images reveal progressively rougher and more heterogeneous topographies, characterized by enlarged microvoids, particle pull-outs, and dispersed filler-matrix interfacial features. Groups G5 to G7 correspond to re-injection moulded composites of ABS reinforced with identical CP concentrations as in G2–G4. These specimens show microstructural features similar in trend to their first injection moulded ABS+CP (G2–G4) counterparts. No changes were observed on the fracture surface of these specimens that would indicate material degradation or interphase delamination due to reprocessing.

However, a noticeable difference in fracture surfaces was observed between virgin ABS and other carbon powder filled ABS. Here, it can be seen that carbon powder particles can serve as a reinforcing element that can locally increase strength, which is why the surface appears rougher and more rugged. As for the comparison between first-injected and re-injected specimens’ fracture surfaces in filled ABS, a more uniform particle arrangement can be observed in re-injection moulded specimens ABS+CP (G5–G7). A possible reason for this is that multiple processing results in multiple mixing and homogenization during the manufacturing process. Moreover, the movement of the barrel screw may have caused the particles to break down into smaller fragments [54], which then dispersed more evenly throughout the matrix, resulting in a more compact structure.

#### 3.4.2. Three-Dimensional-Printed Specimens Fracture Surface Evaluation

**Figure 17:**
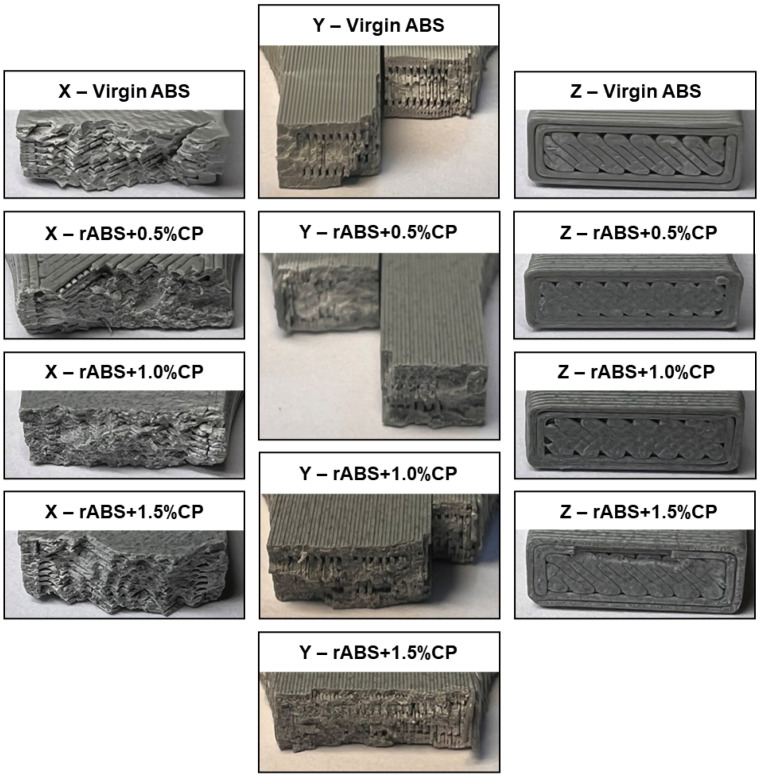
Fracture surface of selected 3D-printed specimens.

At first observation, distinct differences in fracture behaviour can be discerned across the various print orientations, as revealed in Figure 17. Despite employing a 100% infill setting, it is particularly evident in the orientation Z that the internal structure of the specimens is not entirely filled, but with visible voids and unbonded regions present within the printed volume. Such internal discontinuities likely contribute to the observed variability in mechanical performance of the 3D-printed parts. In the X-oriented specimens, a consistent fracture pattern was noted across all CP concentrations, indicating a relatively uniform failure mechanism. Conversely, specimens printed in the orientation Y exhibited complex fracture behaviour, characterized by failure across two planes, including the direction of applied tensile load. This suggests that the interlayer cohesion within the thinner deposited layers is insufficient to effectively transfer the applied stress. However, in rABS+1.5%CP specimens printed in the orientation Y, the presence of carbon powder appeared to enhance the interfacial bonding, as evidenced by improved tensile performance and more cohesive fracture surfaces. A similar reinforcing effect of the added CP was observed in the orientation Z, which is inherently the weakest due to its reliance on interlayer adhesion. Given that the orientation Z has the most pronounced impact on mechanical behaviour under tensile loading, careful consideration must be given during the design phase to avoid geometries that promote such vulnerable configurations in functional components.

Visual examination of the fracture surfaces reveals that the incorporation of 1.5 wt.% carbon powder (CP) into the ABS matrix exerts a beneficial influence on the overall fracture behaviour and interlayer cohesion in 3D-printed specimens. Compared to unmodified or lower CP-loaded counterparts, the specimens containing 1.5 wt.% CP exhibit markedly enhanced morphological features indicative of improved energy dissipation and crack propagation resistance. Consequently, the addition of 1.5 wt.% CP not only reinforces the matrix at the microscale but also contributes to the macroscopic integrity of the printed part by mitigating delamination and promoting cohesive failure across layer boundaries. These findings underscore the potential of CP reinforcement as an effective strategy for tailoring the mechanical performance of polymeric materials in additive manufacturing applications.

## 4. Discussion

The recycling of filled thermoplastics and thermosetting polymers presents considerable challenges, primarily due to economic factors and technical complexities involved in reclaiming materials for subsequent high-value applications. The inherent difficulty arises predominantly from the fillers themselves, as their separation from the polymer matrix is typically complicated. This study examined the possibility of mechanical recycling of carbon powder-reinforced ABS with focus on the tensile testing of recycled products after various production methods: injection moulding, filament production, and 3D printing. In general, the results suggest that the addition of CP has a beneficial influence on tensile properties and confirm that the ABS+CP composite retains its tensile strength and elastic modulus even after multiple reprocessing cycles.

These conclusions of this study are consistent with previous research, which, however, concentrated on pure ABS without fillers. García et al. [49], demonstrated that repeated processing of virgin ABS via injection moulding does not substantially degrade mechanical properties until after the third cycle. This study confirmed similar trends even for carbon powder-filled ABS composites, revealing an enhancement in mechanical performance upon recycling. Specifically, the incorporation of 1.5 wt.% carbon powder (CP) into ABS and subsequent re-injection resulted in approximately 3% increase in tensile strength and a 15% increase in elastic modulus compared to virgin ABS.

The second prominent recycling approach explored in contemporary research, particularly relevant to additive manufacturing, involves fused filament fabrication (FFF) technology. Vidakis et al. [51] previously reported improved mechanical properties of recycled virgin ABS processed via FFF up to the fifth recycling cycle, with optimal performance observed between the third and fifth cycles. However, no research has yet addressed the recycling of carbon powder-filled ABS composites using FFF technology. This highlights a significant gap in the research, which this study aims to fill.

Like the injection moulded specimens, the specimens produced in this research from recycled composites via extrusion and FFF also showed improved tensile properties compared to virgin ABS. Specifically, reprocessed ABS filled with 1.5 wt.% CP exhibited enhanced tensile properties in comparison to virgin ABS in all but one case of the tested 3D-printed orientations (X, Y, and Z). For this filler concentration, the tensile strength showed an average increase of 12% and the elastic modulus an average increase of 27% relative to virgin ABS across all three printing orientations. These observations suggest improved interfacial adhesion and more uniform dispersion of carbon powder upon repeated processing. Such results also confirm the optimal filler concentration within the ABS matrix. Higher filler loadings, exceeding 1.5 wt.%, were not evaluated due to practical difficulties in processing under identical conditions, which would have undermined comparative analyses. Utilizing standardized printing conditions available in Prusa Slicer’s Generic ABS profile facilitated comparability across research efforts. The aforementioned difficulties mainly consist of clogging of the nozzle with filler and imperfect dispersion of the mixture during printing, and thus the need for individual adjustment of printing parameters for each mixture with a filler content exceeding 1.5 wt.%. Given that successful FFF printing requires suitable rheological properties for extrusion through standard nozzles (typically 0.4 mm in diameter), the filler particle size must be sufficiently small to avoid undue resistance. Moreover, hardened nozzles are recommended to mitigate the abrasive effects of fillers, ensuring consistent extrusion and deposition quality.

This study also found that the tensile strength of the extruded filament was, on average, 6% higher than that of the 3D-printed specimens in the Y orientation, which proved to be the strongest among the three (X, Y, and Z). Cress et al. [50] arrived at similar results in their research and attributed them to the inherently imperfect bonding between individual layers during 3D printing, as well as to insufficient material filling, both of which can lead to the formation of microcavities. Similarly, the superior tensile properties observed in the injection moulded specimens among all processing methods can be attributed to the high pressure applied during moulding, which ensures complete filling of the mould and results in a compact, defect-free structure [55,56,57].

**Figure 18:**
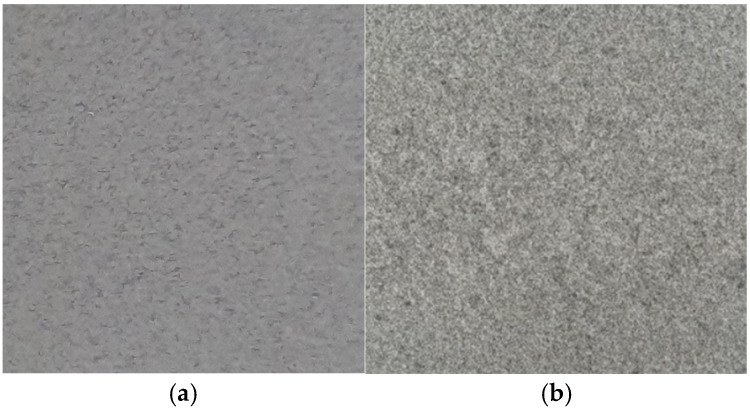
Surface of (**a**). ABS+CP and (**b**). granite.

Overall, this research underscores the feasibility and economic viability of recycling carbon powder-filled ABS composites using readily accessible technologies such as injection moulding and FFF 3D printing. Particularly notable is the suitability of FFF technology for producing structurally and aesthetically enhanced components. Products manufactured through FFF from recycled ABS+CP demonstrated improved mechanical properties compared to virgin ABS and exhibited appealing visual textures reminiscent of granite (Figure 18a), enhancing their potential applicability not only in functional but also in decorative applications.

The present study initially focused on laboratory-based processing of pristine materials, uncontaminated by external agents during prior stages, thereby excluding the confounding effects associated with contaminants. Additionally, this approach eliminated the challenges related to inefficient sorting and collection processes. In this regard, the study simulated an idealized lifecycle scenario for test specimens under controlled laboratory conditions. Nevertheless, future stages of this research must critically evaluate how real-world usage and contamination influence processing and the final mechanical properties of carbon powder-filled ABS at the end of the product lifecycle. In practice, recycled products are often contaminated with foreign substances or undesirable additives that strongly adhere to their surfaces and become incorporated into the polymer matrix during the recycling process, significantly complicating material recovery. These contaminants, often incompatible with the polymer matrix, substantially diminish mechanical properties and compromise the overall performance of the recycled composite. A further complexity lies in the collection and sorting processes for filled polymer composites. Despite their apparent similarity, composites such as carbon powder-filled acrylonitrile-butadiene-styrene (ABS) demonstrate considerable variability due to differences in terpolymer composition and filler modifications. For instance, untreated carbon fibres typically exhibit poor adhesion to the polymer matrix, potentially reducing mechanical performance rather than enhancing it. Hence, mastering the recycling of carbon powder-filled ABS necessitates addressing a multitude of influencing factors that significantly affect both the recycling process and the resulting material properties.

## 5. Conclusions

This study confirms the viability and efficacy of recycling carbon powder-filled ABS composites using injection moulding and FFF 3D printing technologies. Both methods demonstrated the potential to maintain or even enhance mechanical properties of recycled composites compared to virgin ABS. Particularly, the addition of 1.5 wt.% CP was identified as optimal, significantly improving tensile strength and elastic modulus, attributed to enhanced interfacial bonding and improved filler dispersion upon repeated processing. The promising results achieved using FFF technology highlight its suitability for recycling composite materials, offering both mechanical integrity and aesthetically appealing characteristics suitable for various applications.

Future research should extend these findings by examining practical recycling scenarios involving contamination, real-life wear conditions, and environmental degradation effects to fully understand the limitations and potentials of recycled ABS+CP composites. Additionally, further studies are recommended to explore the impact behaviour and fracture toughness of these recycled composites, as their dynamic mechanical properties under impact loading remain underexplored, yet are critically relevant for structural applications. Understanding the energy absorption characteristics and failure mechanisms under rapid loading conditions would greatly enhance the applicability and reliability of recycled ABS+CP composites in diverse industrial sectors, such as automotive and consumer goods, where impact resistance is essential. Comprehensive lifecycle assessments and economic evaluations should also be conducted to establish a complete picture of the environmental and financial benefits associated with these recycling strategies.

## Figures and Tables

**Table 1:** Identification of injection moulded test specimen groups.

Identification of Test Specimen Groups	Group Title
Injection moulded virgin ABS	G1
Injection moulded ABS + 0.5 wt.% CP	G2
Injection moulded ABS + 1.0 wt.% CP	G3
Injection moulded ABS + 1.5 wt.% CP	G4
Re-injection moulded rABS + 0.5 wt.% CP	G5
Re-injection moulded rABS + 1.0 wt.% CP	G6
Re-injection moulded rABS + 1.5 wt.% CP	G7

**Table 2:** Injection moulding parameters.

Processing Conditions	370 S
Injection Rate (cm^3^/s)	28
Injection Pressure (MPa)	80
Holding Pressure (MPa)	60
Holding Time (s)	10
Cooling Time (s)	25
Mould Temperature (°C)	60
**Barrel Temperature Zones**	
Zone 1 (°C)	240
Zone 2 (°C)	245
Zone 3 (°C)	250
Zone 4 (°C)	255
Zone 5 (°C)	260

**Table 3:** Filament extrusion process temperatures.

Barrel Temperature Zones	
Zone 1—hopper (°C)	220
Zone 2 (°C)	230
Zone 3 (°C)	230
Zone 4—nozzle (°C)	240

## Data Availability

The data presented in this study are available on request from the corresponding author.
